# Case Report of Thrombotic Thrombocytopenic Purpura in a Previously Healthy Adult

**DOI:** 10.21980/J8VK9M

**Published:** 2021-01-15

**Authors:** Jessica L Sea, Stephen F Gassner, Jonathan Smart

**Affiliations:** * University of California, Irvine, Department of Emergency Medicine, Orange, CA

## Abstract

This case report details the presentation of a 24-year-old male presenting with headache, palpitations, gingival bleeding, and pallor. The ultimate diagnosis of thrombotic thrombocytopenic purpura (TTP) is a rare but life-threatening condition which is typically hallmarked by thrombotic microangiopathy (TMA) and dysfunctional ADAMTS13 enzymatic activity. This patient displayed classically described findings, including marked anemia, thrombocytopenia, and acutely elevated creatinine as well as a brief period of newly onset word-finding difficulty while in the emergency department. His diminished ADAMTS13 activity level (<5%) and the presence of autoantibodies against ADAMTS13 confirmed a diagnosis of TTP. Fortunately, the patient’s outcome was favorable after treatment with plasmapheresis, prednisone, and rituximab. This case report provides a review of the clinical presentation, diagnostic criteria, potentially confounding differential diagnosis, and expected course of treatment for patients with TTP.

**Topics:**

Thrombotic thrombocytopenic purpura (TTP), ADAMTS13, anemia.

**Figure:**
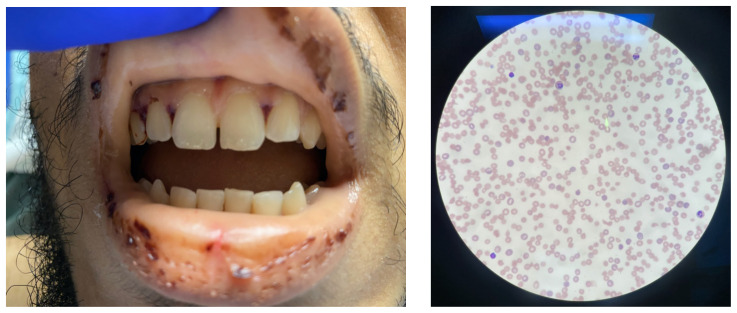

## Introduction

Thrombotic thrombocytopenic purpura (TTP) is a rare, lifethreatening thrombotic microangiopathy (TMA) often occurring in adulthood with a sudden, unpredictable onset.^1^ Deficiency of ADAMTS13 enzyme activity represents the central feature of TTP and is traditionally attributed to either a congenital or acquired condition. Congenital TTP (cTTP) may result from one of more than a hundred well-characterized gene mutations; whereas, acquired or immune-mediated TTP (iTTP) is an acute immunologically mediated episode hallmarked by autoantibodies targeting ADAMTS13. Antibodies against ADAMTS13 have long been thought to be either inhibitory or non-inhibitory, thus limiting the enzyme’s activity or resulting in severe enzyme deficiency, respectively. Recent research suggests both types of antibodies likely function to accelerate clearance of ADAMTS13 leading to diminished or undetectable levels in circulation.^2^

While recent studies have increased our understanding of the mechanism, less is known about specific stimuli that trigger acute episodes of iTTP. Associations have been made between iTTP and connective tissue disorders, pregnancy, certain forms of cancer, and surgeries.^3^–^7^ There have also been a few reported cases of iTTP secondary to severe trauma.^8^ Here, we describe a case of spontaneous iTTP in a previously healthy 24-year-old male that highlights the variability in clinical presentation of iTTP and its unique management, focusing on emergency care. We also discuss the classical clinical manifestations, diagnostic criteria, and therapeutic recommendations, as well as the challenges of each of these.

## Presenting concerns and clinical findings

A 24-year-old male presented to the emergency department (ED) with a chief complaint of headache and palpitations. The patient ranked his headache, which had begun 7 days prior, as 6 out 10 on a pain scale and described it as sharp and constant. He also reported 2–3 days of fatigue, palpitations, skin pallor, and gingival bleeding. He was afebrile, tachycardic (HR=119), and had a blood pressure of 131/68. His SpO^2^ was 100% on room air with a respiratory rate of 18.

## Significant findings

The physical exam revealed globalized pallor of his skin as well as conjunctival pallor. Mucous membranes were found to be dry and pale with dried gingival hemorrhages apparent between teeth (*image*). Additionally, mild hepatosplenomegaly was noted. While in the ED, the patient’s urine was dark reddish-brown, which he then reported had been similarly discolored for the past 7 days.

A complete blood count and metabolic panel revealed an elevated anion gap metabolic acidosis, severe anemia, thrombocytopenia, and acute renal injury. The patient’s blood urea nitrogen (BUN; 27mg/dL) and serum creatinine (1.4 mg/dL) levels were elevated, and his serum HCO_2_ was low (21mmol/L) with an anion gap of 14. The patient’s hemoglobin (4.7 G/DL), hematocrit (14.2%), platelet count (10,000/mcl), and haptoglobin (<30 mg/dL) were all severely decreased while his LDH (1885 U/L) and indirect bilirubin (3.2 mg/dL) were significantly elevated. A peripheral blood smear (*image*) also revealed an abnormally high reticulocyte count (18.7%) and presence of schistocytes. When taken these findings together with the significantly elevated LDH, diminished hemoglobin, and nearly undetectable haptoglobin, a hemolysis picture was supported. Direct antiglobulin test (DAT) was negative; decreasing the likelihood of autoimmune-mediated hemolytic anemia. As prothrombin time (PT; 15.2 sec), partial thromboplastin time (PTT; 30.2 sec), and INR (1.2) were only slightly abnormal, TTP was suspected over disseminated intravascular coagulation (DIC). A PLASMIC score of 6 was obtained, indicating a 72% chance of ADAMTS13 activity <15%.

## Patient course

Presenting initially as neurologically intact, the patient displayed a nonfocal lack of strength that resulted in a slow, but overall normal gait. However, four hours after initial presentation to the ED, the patient developed difficulty with word-finding. He was otherwise neurologically intact, demonstrated by an ability to comprehend and repeat back in his own words what was conveyed to him. Additionally, a repeat head CT at this time of neurological change was unremarkable.

A provisional diagnosis of TTP was made based on the clinical findings and PLASMIC score of 6. The patient was admitted to the ICU and his serum was sent to an external laboratory for confirmation of insufficient ADAMTS13 activity. Due to the life-threatening nature of TTP and urgency for immediate treatment, therapeutic plasma exchange, several units of red blood cell transfusions, and immunosuppressive corticosteroids were initiated immediately. The following day, the patient had returned to his neurological baseline and no longer demonstrated word-finding difficulties. His renal function also significantly improved during this time. Results of the patient’s serum sample showed an ADAMTS13 activity level less than 5% (normal =61% or higher) as well as antibodies against ADAMTS13. He continued to receive daily plasmapheresis and prednisone.

On day three of ICU admission, there was a significant concern of relapse due to the continued presence of ADAMTS13 antibodies; therefore, the patient was administered rituximab. On day six of admission, the patient’s platelet levels normalized to 150,000/mcl. He was administered two further treatments of plasmapheresis. Tapering of steroids was begun while the patient’s laboratory values were monitored closely. After 10 days of admission, the patient was discharged as his hemoglobin and platelet levels remained stable off of plasmapheresis.

In total, he received ten rounds of plasmapheresis, five units of red blood cells, and one dose of rituximab, with the intention of three additional doses in subsequent follow-up. The patient reported feeling well and was discharged with instructions to continue taking pantoprazole (40mg/day) while tapering off of prednisone (60mg) over the following 24 days with a dose reduction of 10 mg every four days. He was also instructed of the need to follow up in clinic for three additional doses of rituximab as well as follow-up labs to monitor ADAMTS13 activity, CBC, and hemolysis.

## Discussion

Thrombotic thrombocytopenic purpura is a rare condition hallmarked by thrombotic microangiopathy (TMA) and dysfunctional ADAMTS13 enzymatic activity. The cleavage enzyme, ADAMTS13, plays a critical role in the regulation of the clotting cascade via its capacity to limit circulating levels of von Willebrand factor (vWF).^9^ Patients with acute iTTP resulting from ADAMTS13 deficiency lack the ability to prevent spontaneous adhesion of vWF. The resulting unregulated aggregation of large vWF multimers leads to subsequent platelet activation and microthrombi formation that occludes systemic microvasculature vital to most major organ functions.^9^ While the introduction of plasmapheresis has dramatically improved survival rates, mortality remains between 7–22% in those who receive standard treatment.^10^–^12^ Delayed diagnosis and treatment have been reported to contribute significantly to this mortality rate and to an increased risk of relapse.^10^,^11^ In a small retrospective study examining TTP-related deaths at six institutions, Colling et al. reported an association between mortality and a delay in the initiation of plasma exchange for more than 24 hours after presentation, highlighting the urgency for rapid diagnosis and immediate treatment.^11^ However, early diagnosis of iTTP can be challenging, as symptoms are often generalized and overlap with various other forms of thrombotic microangiopathies, and the clinical presentation can vary widely between patients.^1^,^9^ Further, the gold standard of clinical tests, serum measurement of ADAMTS13 activity, is not accessible in most hospitals and require physicians to send serum samples to outside labs which often have a turnaround time of 2–3 days.

While early initiation of plasma exchange is critical for treating TTP, differentiating TTP from DIC, hemolytic uremic syndrome (HUS), and heparin-induced thrombocytopenia (HIT) may be challenging. The presence of normal PT, PTT, fibrin degradation product (FDP) may be used in some cases to distinguish TTP from DIC as coagulation is usually intact in TPP.^13^,^14^ Similar to DIC, HUS has several shared characteristics with TTP but typically presents in children whereas acquired TTP more often develops in adults.^13^ Further, HUS also commonly presents with mild to moderate thrombocytopenia and severe renal dysfunction, though there are exceptions to this as well.^13^ Additional laboratory tests measuring ADAMTS13 activity and anti-ADAMTS13 antibodies can be used to confirm TTP. However, these are not optimal diagnostic tools during emergent situations as they are time consuming and not always available. Therefore, prompt diagnosis of TTP is often based on a thorough medical history, physical exam, and routine laboratory results.

Traditionally, diagnostic criteria have centered around the presence of a classic pentad of TTP symptoms: thrombocytopenia, hemolytic anemia, neurological abnormalities, fever, and renal dysfunction.^1^,^9^,^10^ Current recommendations for early diagnosis include the use of a PLASMIC score based on a cohort study identifying several covariates among patients with TTP.^15^ The PLASMIC score is determined by assigning one point for each of the following: platelets < 30 mg/dL, lysis (reticulocytes >2.5%, total bilirubin >2.0 or decreased haptoglobin), absence of active cancer, absence of stem cell or organ transplant, mean corpuscular volume < 90 fL, INR <1.5, and Cr < 2.0 mg/dL.^15^ A score of 4 or below indicates low risk of TTP, and a score of 6 or higher correlates with a high chance of TTP. Our patient had a PLASMIC score of 6, indicating a 72% chance that his ADAMTS13 activity would be less than 15% and thus a high probability of TTP.

In this report, we describe a case of iTTP in a previously healthy 24-year-old man. Acute iTTP episodes have been most commonly associated with risk factors that include a previous diagnosis of an autoimmune disorder, HIV infection, and pregnancy while commonly cited triggers include infection and recent surgery. Based on reports of most patients having more than one risk factor or comorbidity prior to developing TTP, it has been proposed that TTP initiation may be linked to a multifaceted process of triggering an inflammatory response and release of complexed vWF into circulation.^16^ Some of the most commonly cited surgeries associated with TTP are vascular and orthopedic procedures which tend to have high rates of hemolysis and shear stress that may predispose patients to developing TTP.^3^ Consistent with these reports, injuries due to trauma likely also result in the mass destruction of endothelial-lining vessels and red blood cells. It is possible that this may contribute to the rare occurrence of iTPP in patients with severe trauma resulting in hemorrhage and bone fracture.

Interestingly, our patient had none of the risk factors associated with TTP, and his past medical history was unremarkable and without any family history of autoimmune disorders. This underscores the need for additional studies to fully understand the mechanisms by which TTP develops as it could assist in clinical decision making and decrease the time to diagnosis and proper treatment. While TTP is rare, this case highlights the importance of including TTP on the differential for patients with unexplained thrombocytopenia and microangiopathic hemolytic anemia. In particular, patients presenting with neurological symptoms, severe anemia, thrombocytopenia, and acutely elevated creatinine should prompt emergency medicine physicians to investigate for TTP.

## Supplementary Information




