# Supplementary figures and images for: Case Report of Thrombotic Thrombocytopenic Purpura in a Previously Healthy Adult
Source: J Educ Teach Emerg Med. 2021 Jan 15;6(1):V1–4. doi: 10.21980/J8VK9M (PMC10332752; doi:10.21980/J8VK9M)

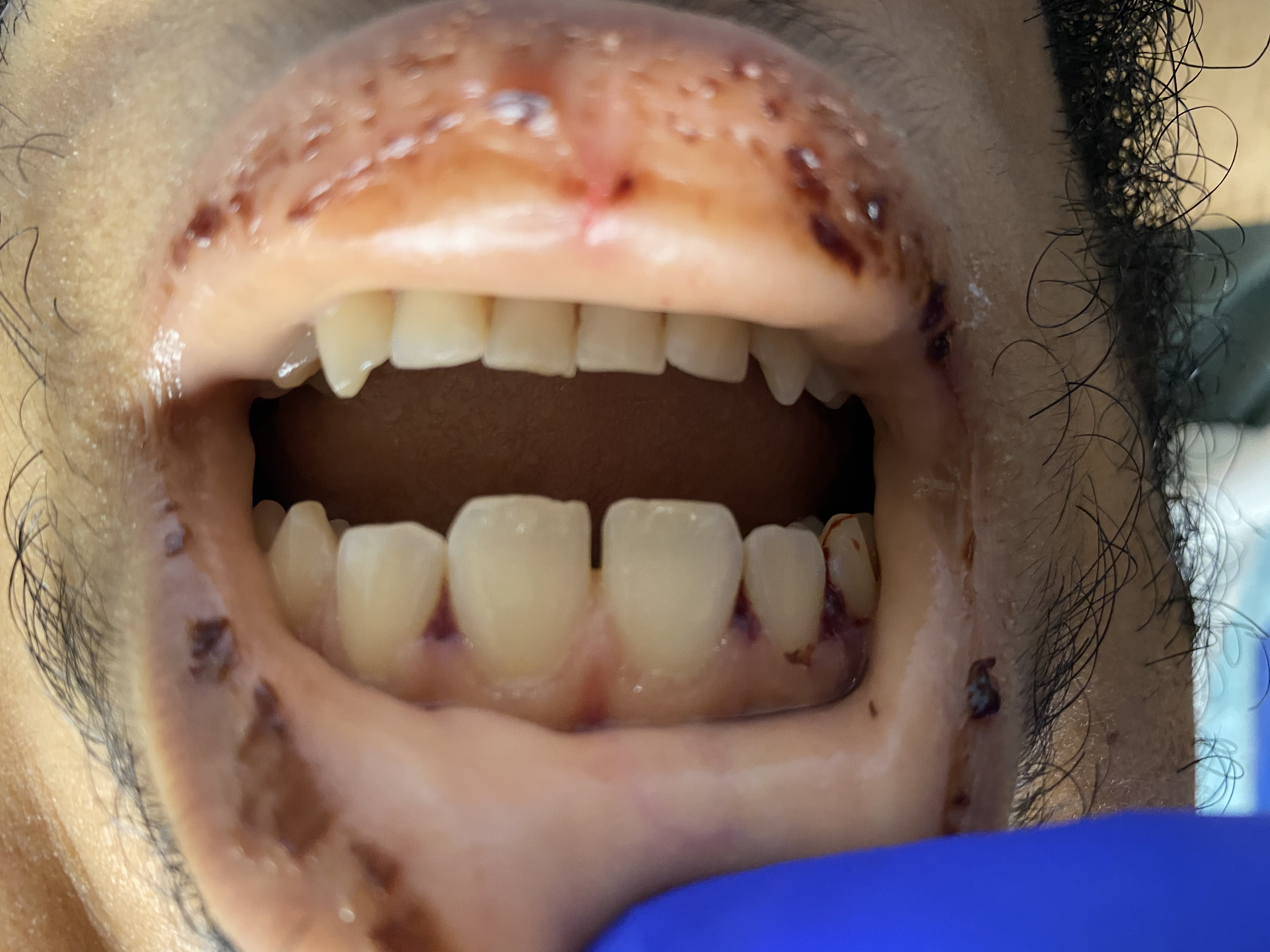

Supplement: Supplementary file 1 [file jetem-6-1-v1-supp1.jpeg]

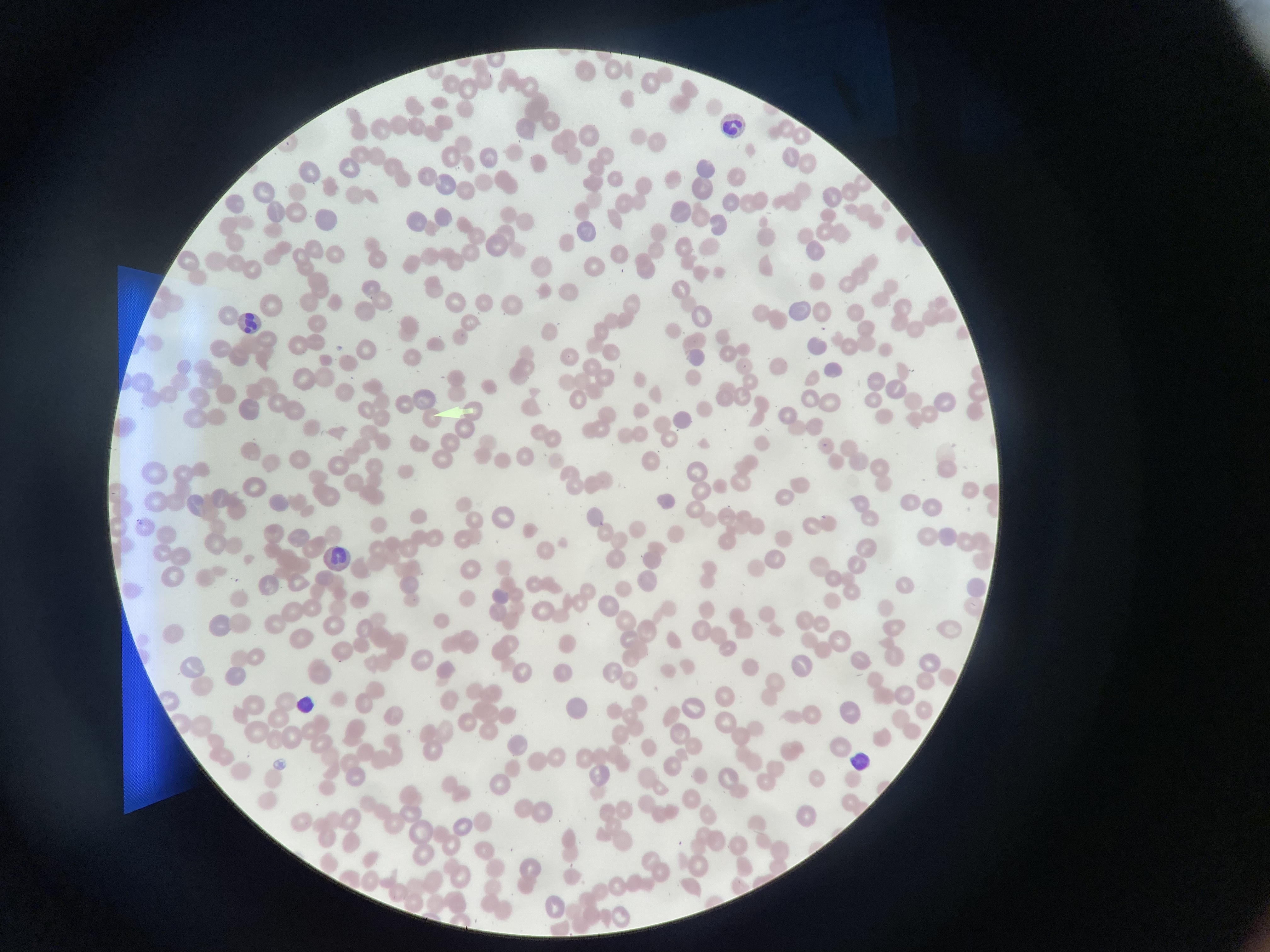

Supplement: Supplementary file 2 [file jetem-6-1-v1-supp2.jpeg]
